# Genomics of circadian rhythms in health and disease

**DOI:** 10.1186/s13073-019-0704-0

**Published:** 2019-12-17

**Authors:** Filipa Rijo-Ferreira, Joseph S. Takahashi

**Affiliations:** 1 Department of Neuroscience, University of Texas Southwestern Medical Center, Dallas, TX 75390-9111 USA; 2 Howard Hughes Medical Institute, University of Texas Southwestern Medical Center, Dallas, TX 75390-9111 USA

## Abstract

Circadian clocks are endogenous oscillators that control 24-h physiological and behavioral processes. The central circadian clock exerts control over myriad aspects of mammalian physiology, including the regulation of sleep, metabolism, and the immune system. Here, we review advances in understanding the genetic regulation of sleep through the circadian system, as well as the impact of dysregulated gene expression on metabolic function. We also review recent studies that have begun to unravel the circadian clock’s role in controlling the cardiovascular and nervous systems, gut microbiota, cancer, and aging. Such circadian control of these systems relies, in part, on transcriptional regulation, with recent evidence for genome-wide regulation of the clock through circadian chromosome organization. These novel insights into the genomic regulation of human physiology provide opportunities for the discovery of improved treatment strategies and new understanding of the biological underpinnings of human disease.

## Background

**Fig. 1:**
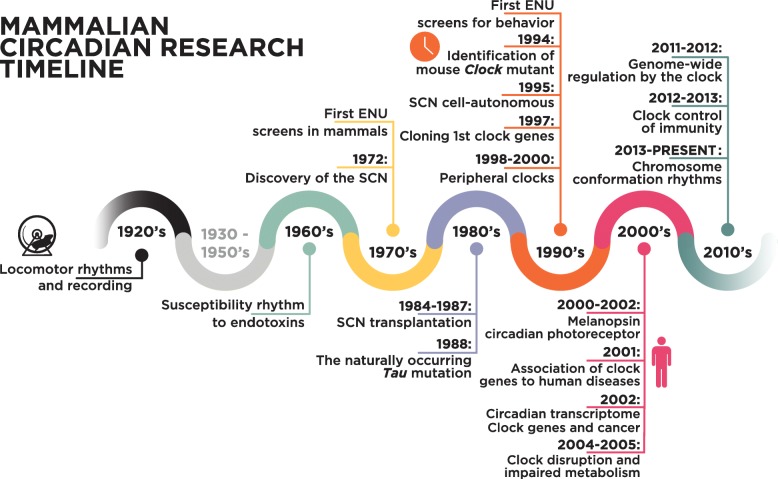
Timeline of major findings in mammalian circadian clock research. *1920s*: first long-term recordings of locomotor rhythms in rats (reviewed in [12]). *1960*: Cold Spring Harbor Symposium on Biological Clocks. First observations that time of day determines susceptibility to endotoxins [13]. *1972*: lesion studies show that the suprachiasmatic nucleus (SCN) of the hypothalamus regulates adrenal corticosterone and drinking behavior rhythms [14, 15]. *Late 1970s and 1980s*: first ENU screens for novel gene identification were performed in mammals [16]. *1984–1990*: identification of the SCN as a master regulator through transplantation experiments [17, 18]. *1988*: a naturally occurring circadian Tau mutation was identified in hamsters [19]. *1990s*: first mammalian ENU screens for behavior, leading to the identification of the first mammalian clock gene, *Clock* [2]. *1995*: circadian rhythms were shown to be cell-autonomous in mammals, being retained in isolated SCN neurons [20]. *1997*: cloning of the Clock gene, which was shown to belong to the bHLH–PAS family of transcription factors. In the same year, the mammalian *Per1* gene was also cloned, both providing entry points for identifying the mechanism of circadian rhythmicity in mammals [3, 8]. *1998–2000*: Discovery of BMAL1/MOP3 as the partner of CLOCK [5, 11], repression by CRY [10] and the Per1/2-Cry1/2 feedback loop on CLOCK:BMAL1 [21]. First descriptions of circadian clocks in the periphery [22, 23]. The cloning of the hamster Tau mutant identified CK1ε as an important kinase regulating the core circadian clock [24]. *2000s*: melanopsin was identified as the circadian photoreceptor in the retina [25–27]. *2001*: first mutation in a clock gene associated with human disease [28]. *2002*: first circadian transcriptomes revealed a significant subset of genes that have cyclic gene expression with a 24-h period [29–31]. *2004–2005*: association of mutations in clock genes with impaired metabolism [32, 33]. *2011*: peroxiredoxin cycles reported to be independent of transcription [34]. *2011–2012*: detailed descriptions of genome-wide regulation by the clock [35–38]. *2012–2013*: major advances in our understanding of the clock control of immunity [39–42]. *Present day*: a new layer in our understanding of genome-wide regulation by the clock through circadian chromosome organization is emerging [43–45]. ENU, N-ethyl-N-nitrosourea

Circadian rhythms are driven by an internal timing system regulated at the transcriptional level that gives rise to gene networks that oscillate with a 24-h cycle. Within these networks are clock genes that control rhythms in physiology and behavior. Interestingly, the circadian clock genes were among the first genes to be identified as controlling behavior. Following studies by Konopka and Benzer [1], who identified the first circadian mutant—*period*—in fruit flies, a forward-genetic behavioral screen was implemented in mice. Through this screen, the first circadian mutant mouse was identified [2], followed by the cloning of the first mammalian circadian gene, *Clock* [3]. Research into the mechanisms of mammalian circadian rhythms then exploded, with many additional genes added to the clock core loop [4–11] (Fig. 1). Since then, it has become clear that the circadian system plays an overarching role in regulating human physiology [46]. Recent studies have provided further lessons on how disruption of circadian rhythms is associated with sleep disorders [47, 48], cancer [49, 50], susceptibility to infections [51, 52], metabolic syndrome [53], Alzheimer’s disease [54], and aging [55]. There is also some indication that, in addition to controlling circadian gene expression, clock genes can influence other cellular functions in a non-circadian manner [56].

This review focuses on the most recent advances in mammalian circadian rhythms research, highlighting novel techniques and explaining the importance and implications of these research findings for human disease, translational research, and medicine. We discuss a number of modern genomics approaches to the study of circadian rhythms, such as the evaluation of chromatin dynamics and gene regulation. Owing to the circadian functions that are common to these diseases, another factor that we highlight is the opportunity to intervene using the timed administration of drugs (chrono-pharmacology) or by targeting clock components. Indeed, as we discuss throughout this review, there may be great benefits to considering circadian timing in the treatment of metabolic disorders, cardiovascular disease, and cancer [53, 57, 58].

## Current view of the mammalian molecular clock

**Fig. 2:**
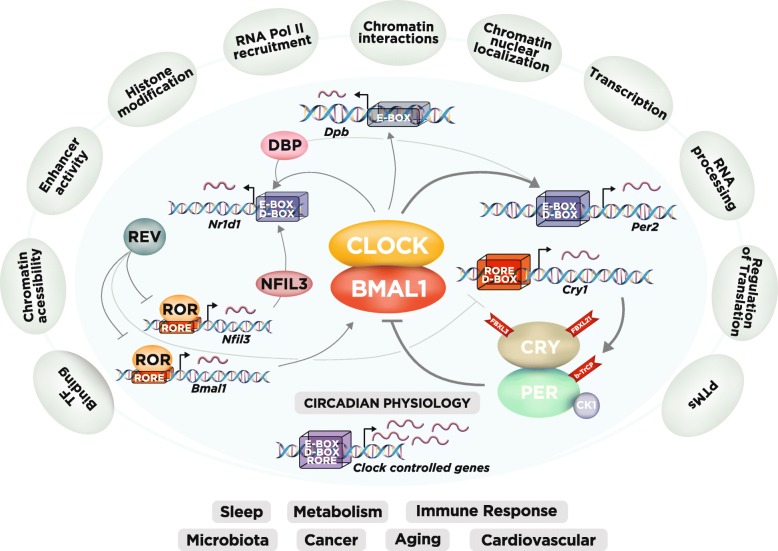
The circadian gene network and layers of genome-wide regulation in mammals. At the core of the network, the transcription factors CLOCK and BMAL1 activate the *Per1*, *Per2*, *Cry1*, and *Cry2* genes (here we show *Per2* and *Cry1* as examples), whose protein products (PER and CRY) repress their own transcription. The PER and CRY proteins are post-translationally regulated by parallel E3 ubiquitin ligase pathways (FBXL3 and FBXL21 for CRY and β-TrCP for PER), with PER levels being also regulated by CK1. CLOCK and BMAL1 also regulate the expression of the *Nr1d1/2* genes, which encode the nuclear receptors REV-ERBα/β, respectively. These nuclear receptors rhythmically repress the transcription of *Bmal1* and *Nfil3*, two genes that are activated by retinoic acid-related orphan receptor-α/β (RORα/β). In turn, NFIL3 together with D-box binding protein (DBP), as well as CLOCK and BMAL1, regulate a rhythm in the REV-ERBα/β nuclear receptors. These three interlocked transcriptional feedback loops regulate the majority of cycling genes, leading to rhythms in various different physiological systems, from sleep to metabolism and aging (*bottom* of figure). Note that the E- and D-boxes and the RORE-binding regions are in *cis* upstream at the promoter; however, they are represented here as a *stacked box* for simplicity. Recent work has identified additional levels of regulation of circadian gene expression (outer layer of regulation in the figure), including rhythmic histone modifications, RNA polymerase II (Pol II) recruitment, circadian chromosomal conformation interactions and post-translational modifications (PTMs). Please refer to Table S1 for many of the studies that allowed the external regulatory layers to be added to the comprehensive view of the clock

The circadian clock in mammals is cell-autonomous and depends on transcriptional autoregulatory feedback loops (Fig. 2). Circadian rhythms are also tuned at the post-transcriptional [59] and post-translational levels [60], although gene transcription remains vital for making the clock ‘tick’. Genome-wide approaches (Additional file 1: Table S1) have found that rhythmic transcription is accompanied by rhythmic transcription factor binding and histone modifications in enhancer regions [61], as well as by circadian recruitment of RNA polymerase II (Pol II) to DNA [35, 36, 62]. An additional layer of regulation involves chromosome organization, with interactions of active and repressive chromosomal domains undergoing circadian oscillations [63–67].

Recently, studies in mouse tissues have greatly enhanced our understanding of the circadian regulatory mechanisms for rhythmic transcription [43–45, 68, 69]. Sobel et al. [68] characterized the chromatin accessibility landscape by mapping DNase I hypersensitive sites (DHSs) in mouse liver across 24 h. DHS sites reflect open chromatin and their occupation of transcription start sites (TSSs), enhancers, and silencers mean that they are hallmarks of regulatory DNA. In this study, the authors found that 8% of 65,000 DHSs cycled with a 24-h period, in phase with Pol II binding and histone 3 lysine 27 acetylation (H3K27ac) marks, suggesting that regulatory elements within DHSs control rhythmic transcription [68]. Two additional studies have further advanced our understanding of chromatin interactions [43, 44]. Mermet et al. [43] utilized circular chromosome conformation capture sequencing (4C-seq) to explore the three-dimensional chromatin interactions of a locus of interest with other genomic regions (one-to-all). They examined the TSSs of the clock repressor gene *Cryptochrome 1* (*Cry1*) and of a liver-specific clock-controlled gene, *Gys2* (*Glycogen synthetase 2*), which encodes the rate-limiting enzyme in hepatic glycogen synthesis. These genes show rhythmic transcription with opposite phases, allowing the authors to correlate their chromatin interaction profiles with their gene transcription regulation. The authors found that chromatin contact with such regions increases at the time of the day when the corresponding gene has its peak expression. Strikingly, abrogation of an enhancer that is rhythmically recruited to the *Cry1* promoter leads to a shortened period of locomotor activity, suggesting that such interacting loops are necessary for the modulation of rhythmic behaviors [43]. Together, these studies show that rhythmic modulation of chromatin conformation adds an important layer of control over circadian gene transcription (Fig. 2).

Despite these genome-wide advances, our understanding of circadian regulation at the protein level is much more limited, mostly because of the difficulty of quantitative assessment of the proteome [70, 71]. Recent technological advances have allowed quantification of the circadian proteome, the nuclear proteome [72], and the phospho-proteome [73]. These studies revealed the rhythmic presence of about 500 proteins (~ 10%) in the nucleus that are components of nuclear complexes involved in transcriptional regulation, ribosome biogenesis, DNA repair, and the cell cycle [72]. Strikingly, more than 5000 (~ 25%) phosphorylation sites are rhythmic, far exceeding rhythms in protein abundance (phosphorylation is an example of post-translational modification (PTM); Fig. 2). Overall, recent studies have vastly enhanced our understanding of the genome-wide reach of the molecular clock and how it is regulated.

## Circadian control of sleep

### Human circadian sleep disorders and their genetic causes

**Fig. 3:**
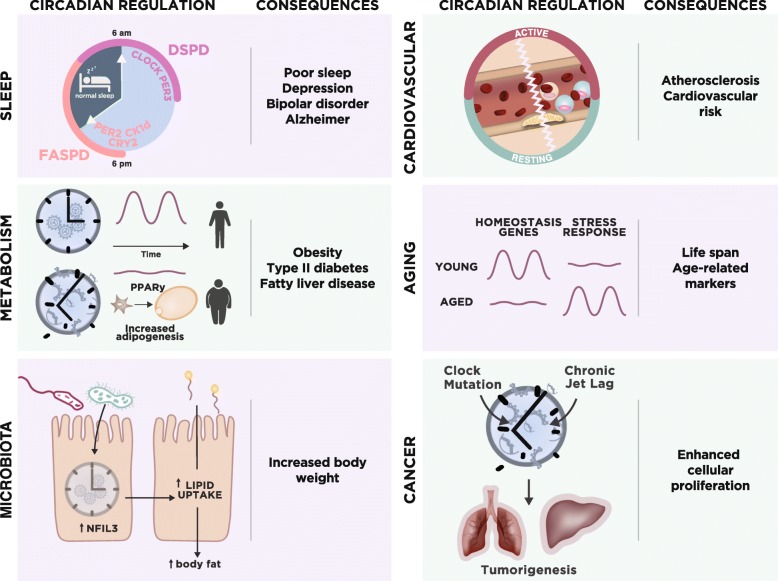
Highlights of circadian regulation across different physiological systems. *Sleep*: overview of circadian disruptions that directly modulate the timing and quality of sleep in humans [28, 47, 48, 76, 77] and the consequences of poor rhythms [78–80]. The outer layers represent the time at which individuals who have either familial advanced sleep phase disorder (FASPD) or delayed sleep phase disorder (DSPD) usually sleep. *Metabolism*: the integration of corticosterone rhythmic signaling by PPARγ in adipogenesis [81] and the metabolic consequences of disrupted rhythms [32, 33]. *Cardiovascular system*: neutrophils and monocytes adhere to atherosclerotic plaques (represented as the *yellow mass* in the inner side of the blood vessel) during the transition from the active to the resting period [57]. Clock disruption also impacts the vascular system [82]. *Aging*: reprogramming of circadian gene expression in stem cells in aging [83] and the consequences of poor rhythms [84]. *Microbiota*: gut microbiota upregulate NFIL3 levels, which modulate lipid uptake and body fat [85]. *Cancer*: disruption of the circadian clock leads to enhanced cell proliferation and tumorigenesis [49, 50]

In humans, mutations in circadian clocks have been associated with circadian rhythm sleep disorders. Familial advanced sleep phase disorder (FASPD) is a circadian rhythm sleep disorder with habitual sleep times that are earlier than the societal norm. The first identified cause of FASPD was a missense mutation (S662G) in the *PER2* gene [28]. Casein kinases Iδ and Iε (CKIδ/ε) regulate levels of PER2 by phosphorylation-mediated degradation and cellular localization (Fig. 2). The S662G mutation appears to be in the CKIε binding site, which causes hypophosphorylation by CKIε in vitro. Deficient phosphorylation of PER2 in the cytoplasm may impair its degradation and lead to nuclear accumulation [28, 74]. FASPD has also been linked to a missense mutation (T44A) in the human *CKI*δ gene. This mutation leads to reduced kinase activity in vitro and to a shorter circadian period in mice [75]. Recently, Hirano and colleagues [48] described another missense mutation in the human *CRY2* gene that is associated with FASPD. The alanine to threonine mutation (A260T) in CRY2 is located in its flavin adenine dinucleotide (FAD) binding domain. Such mutation increases the affinity of FAD for the E3 ubiquitin ligase FBXL3, thus promoting its degradation (Fig. 3).

A less understood, but more common, type of circadian rhythm sleep disorder, with an estimated prevalence of almost 10% in the general population, is delayed sleep phase disorder (DSPD; reviewed in [86]). It is characterized as a type of insomnia with inconsistent and delayed sleep onset and offset times compared to the societal norm. Familial cases of DSPD have been described, suggesting that Mendelian inheritance of DSPD may exist with polymorphisms in the *CLOCK* or *PER3* genes (reviewed in [87]). Patke et al. [47] recently reported a hereditary form of DSPD that is associated with a mutation in *CRY1*, in the 5′ splice site of exon 11, which leads to skipping of exon 11 and ultimately to an in-frame deletion of 24 residues in the C-terminal region of CRY1. Such changes lead to an enhanced affinity of this repressor for the circadian activator proteins CLOCK and BMAL1, which lengthens the period of circadian molecular rhythms [47] (Fig. 3). Together with the studies on FASPD, human genetics has helped to unravel some of the circadian drivers of sleep; nevertheless, there is still much to be learned about how these signals permit or inhibit sleep cycles. With biobanks increasing in size and the advent of direct-to-consumer genetic testing provided by companies such as 23andMe, the genetic information available about populations has increased. Taking advantage of such data, multiple loci have been associated with chronotype, that is, whether people describe themselves as morning people (‘larks’) or as evening people (‘owls’) in terms of sleeping habits. Among the genetic hits associated with chronotype are known clock genes, such as *PER1*, *CRY1*, and *BMAL1* [88].

### Circadian genomics and sleep regulation

In mice, a recent study has suggested a novel link between metabolism and sleep regulation. Salt-inducible kinase 3 (SIK3) is a serine-threonine kinase in the AMP-activated protein kinase (AMPK) family that is known to act as an energy sensor. Not surprisingly, *Sik3*^*−/−*^ mice exhibit severe metabolic symptoms, such as hypolipidemia and hypoglycemia, many dying immediately after birth [89]. SIK3 impacts the stability of the PER2 protein, but unlike *Per2* mutants [6], *Sik3*^*−/−*^ mice have a longer circadian period based on activity and, perhaps as a result of this, show a 6-h phase delay in their oxygen consumption rhythm. Curiously, a point mutation in *Sik3* that causes a profound increase in total sleep time has been identified in a forward-genetics screen [90]. Whole-exome sequencing revealed that the mutation led to the skipping of exon 13, encompassing the protein kinase A (PKA) recognition site in SIK3. However, in contrast with the phenotype observed in the *Sik3*^*−/−*^ mice, there was no effect on circadian period length as assessed by wheel-running behavior under constant darkness [90]. Taken together, it seems that (at least in mice) SIK3 has a critical role in the regulation of sleep and circadian rhythms.

## Circadian control of metabolism

Driven by the circadian clock, a regular daily pattern of eating and fasting maintains normal circadian physiology. However, recurrent disruption of daily activity–rest rhythms, and thus feeding patterns (as occurs in shift workers), is associated with metabolic syndrome [91]. Genetic disruption of the circadian clock also predisposes rodents to metabolic disease [32, 33]. The clock controls metabolism directly by driving transcriptional programs for certain metabolic pathways. For example, CRY1 suppresses hepatic gluconeogenesis during fasting through the regulation of cAMP/CREB signaling, the rhythmic repression of the glucocorticoid receptor gene, and the suppression of nuclear FOXO1 that, in turn, downregulates gluconeogenesis [92–94]. Another clock repressor, PER2, controls lipid metabolism by direct regulation of peroxisome proliferator-activated receptor gamma (PPARγ) and mitochondrial rate-limiting enzymes [95, 96]. The nuclear hormone receptors, REV-ERBs, also directly regulate the transcription of several key rate-limiting enzymes for fatty acid and cholesterol metabolism [97] (reviewed in [98]). Disruption of CLOCK and BMAL1 has also been associated with obesity, hyperinsulinemia, and diabetes [32, 33, 99, 100]. The circadian posttranscriptional regulator Nocturnin also controls lipid and cholesterol metabolism [101]. Recently, an atlas of circadian metabolic profiles across eight tissues revealed temporal cohesion among tissues, whereas nutritional challenge (a high-fat diet) impacted each tissue differentially [102]. In addition to direct modulation of mammalian metabolism, indirect control by the clock occurs through its regulation of behavior, food intake, and the oscillation of hormones such as insulin, glucagon, peptide YY, glucagon-like peptide 1, corticosterone, leptin, and ghrelin (reviewed in [103]). Although we know much about the circadian clock’s control of metabolism, the mechanisms behind this control are far from understood [104]. How nutritional challenges dysregulate the clock and how clock disruption increases adipogenesis remain open questions in the field. However, recent studies have contributed to our understanding of such complex phenomena.

### Dietary influences on circadian enhancers

In recent years, time-restricted feeding has revolutionized dietary restriction protocols. Body weight increases are kept to a minimum even when animals are placed on high-fat and/or high-fructose diets by simply restricting food ingestion to an 8–12-h window [105, 106] (reviewed in [107]). The time during which food is consumed should be in sync with the animals’ circadian rhythms, as misalignment leads to metabolic dysfunction [108–111]. In addition, nutrient-sensing neurons (AgRP) experience daily rhythms in response to leptin [112]. The nutritive environment itself appears to impact feeding behavior and imposes dramatic changes in circadian gene expression in diet-induced obesity (DIO) models [113, 114]. Recently, Guan et al. [53] showed that one of these DIO-related changes is the development of newly rhythmic oscillations of the lipogenic transcription factor sterol regulatory element-binding protein (SREBP), which regulates fatty acid synthesis and oxidation, and of the peroxisome proliferated activated receptor alpha (PPARα), a major regulator of fatty acid oxidation. This is likely to be a consequence of circadian rhythms that are evoked at the enhancers of genes that are not normally rhythmic [53]. Moreover, a PPARα agonist (WY-14,643) is more efficacious in lowering lipids when administered at the circadian peak of PPARα expression. This suggests benefit in considering chrono-pharmacological interventions for the treatment of metabolic disorders.

In search of compounds that modulate the circadian system, an earlier study utilized cell-based circadian reporter assays for high-throughput screening of 200,000 synthetic small molecules. This study revealed compounds that both lengthen and shorten period in both central and/or peripheral clocks [115] (reviewed in [116]). From another more recent screen, nobiletin (NOB), a natural polymethoxylated flavone, was identified as a clock amplitude-enhancing small molecule. In mice with metabolic syndrome caused by DIO or by genetic disruption (*db*/*db* obese mice), NOB augments energy expenditure and locomotor activity in a *Clock* gene-dependent manner, while also strongly blunting body-weight gain, lowering fasting glucose levels, and improving glucose tolerance and insulin sensitivity. However, these beneficial effects of NOB are absent in DIO *Clock* mutants [117], suggesting the potential for pharmacological modulation of metabolism through the enhancement of circadian rhythms. These results, together with those of the other studies on DIO, clearly show that the nutritional environment can have drastic effects on circadian rhythms.

### Adipocyte differentiation

Glucocorticoids and other adipogenic hormones are secreted in mammals in a circadian manner. In addition, high-resolution automated sampling has allowed the identification of ultradian glucocorticoid cycles of approximately 1-h period, and with higher amplitude coinciding with the onset of circadian activity [118]. Loss of glucocorticoid circadian oscillations correlates with obesity in humans, but how do hormone dynamics affect adipocyte differentiation? In a compelling quantitative study, Bahrami-Nejad et al. [81] recently showed that adipocyte differentiation does not progress under normal circadian hormonal cycles. Instead, differentiation is induced if the period of the pulses shortens or if the hormonal signal is flat or continuously elevated [81]. Aberrant glucocorticoid profiles may be caused by abnormal feeding or sleep cycles, long-term glucocorticoid hormone treatment, chronic stress, or metabolic syndrome [119] (Fig. 3). When daily glucocorticoid oscillations are flattened, there is an increase in the mass of subcutaneous and visceral fat pads in mice [81]. This adipocyte differentiation appears to be linked to PPARγ, which acts as a filter of circadian hormonal stimuli. Using these studies as a jumping off point, it will be exciting to find out how ultradian rhythms of glucocorticoids are integrated and what additional circadian factors are critical for regulating adipogenesis.

### Autophagy and circadian rhythms

A recently described link between circadian clocks and autophagy unravels the previously unappreciated role of this degradation pathway in recycling circadian proteins. Autophagy, which degrades cytoplasmic contents in lysosomes, also degrades the repressor CRY1. As mentioned previously, CRY1 suppresses hepatic gluconeogenesis. Toledo et al. [120] investigated the role of autophagy in the regulation of the liver clock and glucose metabolism. They found that the timely degradation of CRY1 by autophagic pathways allows glucose production [120]. Interestingly, obesity increases the autophagic degradation of CRY1, leading to higher glucose production and higher blood-sugar levels. By contrast, loss of autophagy leads to the accumulation of CRY1 and therefore disrupts the clock [120]. These results further highlight that the regulation of clock rhythmicity is itself complex and intertwined with central processes and molecules in our cells. The mechanisms that define the diurnal window of autophagy within cells and the specific timing of the autophagic degradation of CRY1 remain puzzling. These findings also beg the question: what other central processes in cells involve the circadian system? We believe it is likely that many additional functions of circadian rhythms will be uncovered.

## Circadian control of the immune system

Dramatic temporal variation in sensitivity to endotoxins between morning and evening was first discovered in the 1960s [13]; but only in the past decade have major inroads been made in our understanding of clock control over the immune system (Fig. 1). Circadian clock control impinges upon many aspects of the immune response, from the trafficking of immune cells, to the activation of innate and adaptive immunity, to host–pathogen interactions. There have been thorough reviews of these topics [121], so instead we highlight the most recent findings.

### Immune cell trafficking

Cells of the innate immune system, such as neutrophils and monocytes, exhibit circadian patterns of migration from the blood to tissues [122]. Furthermore, T and B lymphocytes, which are cells of the adaptive immune system, were also recently shown to exhibit strong circadian oscillations in the blood, with their numbers peaking during an organism’s resting phase. This rhythmicity continues as lymphocytes are trafficked to the lymph nodes [123–125], with lymphocyte homing to lymph nodes peaking at activity onset, and cells leaving the tissue during the resting period. Using lineage-specific genetic ablation of circadian clock function, Druzd et al. [125] demonstrated that the periodic presence of lymphocytes in the lymph nodes is dependent on the rhythmic expression of promigratory factors on lymphocytes. The rhythmic trafficking of immune cells has also been associated with chemokine receptor CXCR4 expression and is regulated by glucocorticoids [126]. A recent study sheds light on the role of the Interleukin-7 receptor (IL-7R) in this process. IL-7R, whose signaling promotes the proliferation, survival, and differentiation of T cells, has a putative glucocorticoid response element (GRE) on its enhancer; thus, in the presence of glucocorticoids, IL-7R transcription is activated [127]. Intriguingly, glucocorticoids induce IL-7R with a diurnal rhythm, thereby increasing CXCR4 expression and supporting T cell survival and recruitment to various tissues. Moreover, the diurnal variation in T cell distribution enhances immune responses to soluble antigens and systemic bacterial infection at night [124]. Taken together, these findings suggest that cells of the immune system are capable of responding to circadian cues to maximize their ability to respond to infection.

### Clock regulation of innate and adaptive immunity

What role do clock proteins play in regulating the immune system? Several interesting studies have examined the interplay between clock proteins and immune system function [121, 128]. At present, the anti-inflammatory effects of BMAL1 and REV-ERBα are the best understood [39]. Lineage-specific ablation of *Bmal1* in macrophages, the primary effector cells of the innate immune system, eliminates the rhythmic cytokine storm response to endotoxins [39]. It also leads to the abolition of daily protection against sepsis that naturally occurs in mice during the early rest phase [122]. This may be related in part to the regulation of *Bmal1* expression by the microRNA miR-155 in myeloid cells. Endotoxins repress BMAL1 through the targeting of miR-155 to seed sequences in the 3′ untranslated region of *Bmal1*. Thus, the induction of proinflammatory miR-155 correlates inversely with levels of BMAL1. In wild-type mice, BMAL1 inhibits miR-155 induction and protects mice from lipopolysaccharide (LPS)-induced sepsis [129].

Recently, Sutton and colleagues [130] shed some light on how circadian disruption may be linked with an increased incidence of autoimmune disease. Using an autoimmune disease model of multiple sclerosis, the authors show that loss of myeloid BMAL1 creates an inflammatory environment in the central nervous system (CNS) through the expansion and infiltration of IL-1β-secreting monocytes. The result is an increase in pathogenic T lymphocytes, which may contribute to the neuroinflammation and demyelination observed in this disease model [130]. These studies highlight the complexity of immune response coordination between innate and adaptive immune cells and a layer of regulation by the circadian clock.

An additional piece of the puzzle of how the clock regulates the immune response in macrophages is the fact that BMAL1 controls the levels of the antioxidant-encoding gene *Nrf2*, by directly binding an E-box in its promoter in myeloid cells [131]. In macrophages, reactive oxygen species (ROS) promote the production of the cytokine IL-1β via stabilization of HIF-1α [132], which induces the expression of downstream proinflammatory molecules [133]. On the other hand, NRF2 is a transcription factor that protects cells against oxidative damage. Early et al. [131] showed that activation of NRF2, by either genetic or pharmacological methods, rescues the proinflammatory phenotype of *Bmal1*^−/−^ macrophages. These findings suggest a role for the molecular clock in regulating NRF2 in innate immune cells to control the inflammatory response [131]. Despite our increasing understanding of how the clock modulates immune responses, further studies are required to elucidate fully the role of circadian rhythms in immune surveillance and activity.

### Host–pathogen interactions

Many studies have shown that the outcome of an infection (whether bacterial, viral, or parasitic) depends on the time of day at which the infection is initiated [40, 51, 52, 134]. For example, *Salmonella enterica subsp. enterica* serovar Typhimurium (*S. typhimurium*) levels are higher following an infection during the rest phase when compared with infection initiated in the middle of the active phase in mice. This difference is dependent on a functional copy of CLOCK [40]. Similarly, time-of-day of host infection influences virus progression both in live mice and in individual cells. Viral infections of herpes, influenza A, and respiratory viruses of the *Paramyxoviridae* family are enhanced when host circadian rhythms are abolished by disrupting the *Bmal1* gene [52, 135]. *Bmal1*^−/−^ mice that were intranasally infected with the respiratory syncytial virus (RSV) had a higher viral load than wild-type mice [135]. Interestingly, Ehlers et al. [136] found that the misalignment of circadian rhythms through chronic jet lag exacerbates acute viral bronchiolitis caused by Sendai virus (SeV) or influenza A virus in mice [136]. Notably, the authors also showed that in humans, respiratory tract expression of most clock genes (*BMAL1*, *NPAS2*, *PER2*, *DBP*, and *NR1D1* [*REV-ERB α*]) is reduced in adult asthma patients.

Parasite infection also depends on the timing of the host circadian cycle. *Leishmania* parasite burden is circadian in nature, and *Bmal1* in non-lymphocyte immune cells (monocytes) is responsible for modulating the magnitude of *Leishmania* infection [51]. Similar findings were described for the intestinal parasitic helminth *Trichuris muris*, with mice infected at the start of the active phase showing delayed resistance to infection. Remarkably, this response pattern appears to be shifted with daytime-restricted feeding. Cell-lineage-specific genetic ablation of *Bmal1* in antigen-presenting dendritic cells (DCs) in vivo also leads to a loss of time-of-day dependency of helminth expulsion from the body, the result of the resistance to infection [134]. Therefore, the circadian clock (with focus to date mainly on BMAL1) can regulate cellular immunity against bacteria, viruses, and parasites.

Infections or the resulting inflammation can also disrupt the circadian clock by dramatically decreasing the amplitude of circadian rhythms. This has been seen in infections with the causative agents of Chagas disease (*Trypanosoma cruzi*) [137], sleeping sickness (*Trypanosoma brucei*) [138], and malaria (*Plasmodium chabaudi*) [138]. Such downregulation of the expression of clock genes appears to be a consequence of the massive immune response to invasion, as it has also been demonstrated that proinflammatory cytokines are able to decrease the amplitude of rhythms in vitro. Moreover, this type of immune response also alters the animal’s behavior, reproducing what is known as ‘sickness-like behavior’ [139]. Recently, our own study of sleeping sickness (a disease that is almost always fatal if left untreated) concluded that the parasite may disrupt the sleep of patients through the modulation of their circadian clocks. This dysregulation appears to be caused, at least in part, by a systemic signal (possibly secreted by the parasite or possibly a host molecule that is produced in response to infection) that is responsible for shortening the circadian clock period [138]. In summary, although mostly understood from the side of the host’s immune system (and perhaps metabolism), host–pathogen interactions are also subjected to circadian modulation. It is also likely that the circadian rhythms of the pathogens play a role [140]. Further studies are needed to understand these interactions fully.

### Commensal microbiota and circadian rhythms

The metabolic interactions between the gut and its microbiome have been a major research focus in the past decade, and both the host and microbiota rhythms seem to affect one another. Disruption of clock genes in the host abolishes rhythms in the abundance of certain microbiota [141], which seem to be restored upon time-restricted feeding [141, 142]. On the other hand, the absence of gut microbes perturbs the expression of circadian clock genes in the mouse liver [143]. Thaiss et al. [142] showed that the intestinal microbiota in mice undergoes rhythmic fluctuations in its biogeography and metabolome patterns. Recently, Wang et al. [85] found that body composition is regulated by the gut microbiota via the transcription factor NFIL3. This is a circadian basic leucine zipper transcription factor that is expressed in immune cells (Fig. 2). Its levels also oscillate diurnally in intestinal epithelial cells and the rhythms are enhanced by microbiota, as *Nfil3* expression is reduced in germ-free animals. Curiously, the authors found that epithelial-cell-specific *Nfil3* knockout mice were resistant to DIO, and that epithelial NFIL3 controls the expression of a circadian lipid metabolic program and regulates lipid absorption in intestinal epithelial cells (Fig. 3). A recent study also showed that the circadian clock in intestinal cells (focusing on group 3 innate lymphoid cells, ILC3s) is important in regulating susceptibility to bowel infection and lipid metabolism [144]. Altogether, these studies have added a new layer of complexity to the notions of mammalian circadian rhythms and of how the commensal microbiota play a role in homeostasis and body composition.

## Circadian rhythms in the cardiovascular system

Cardiovascular complications have higher incidence in the morning. Many different studies have connected the clock with cardiovascular function, including daily variation in blood pressure, and even response to aspirin [82, 145, 146]. Some studies suggest that pharmacological targeting of REV-ERB decreases atherosclerotic plaque burden in mice [147]. On the other hand, other studies suggest that deletion of *Bmal1* in myeloid cells increased monocyte recruitment and atherosclerosis lesion size [148]. A recent study has shed light on a mechanism that may contribute to this phenomenon. The adherence of myeloid cells to microvascular beds peaks during the early active phase, which appears to be a consequence of peak cell recruitment to atherosclerotic lesions 12 h earlier [57]. Winter et al. [57] showed that both the upregulation of cell adhesion molecules during the active phase by endothelial cells and the presence of immobilized chemokines (emitted by either endothelial cells or myeloid cells) on arterial vessels attract leukocytes into atherosclerotic lesions. Thus, the chemokine CCL2 (C-C motif chemokine ligand 2) and its receptor CCR2 (C-C motif chemokine receptor 2) are at the core of this daily pattern of leukocyte migration and adhesion to the lesions. Importantly, the authors found that timed pharmacological CCR2 neutralization caused inhibition of atherosclerosis without disturbing microvascular recruitment, providing a proof-of-principle treatment schedule for chrono-pharmacological intervention in atherosclerosis (Fig. 3).

Loss of *Bmal1* results in an acceleration of aging and a shortened life span in mice [84]. The cardiovascular system is among the systems affected by aging, with *Bmal1*^−/−^ mice being predisposed to developing atherosclerosis. Using an inducible knockout (iKO), Yang et al. [149] tested whether these age-related phenotypes remained if mice lost BMAL1 as adults. They found that both *Bmal1*^−/−^ and iKO models exhibit markers consistent with accelerated aging (ocular abnormalities and brain astrogliosis), behavioral disruption, and transcriptional dysregulation. This is consistent with the fact that conditional ablation of the pancreatic clock still causes diabetes mellitus [99]. However, some other biomarkers of aging, including premature death in *Bmal1*^−/−^ mice, were not replicated in the iKOs [149]. Among those, the predisposition for atherosclerosis appears to be reversed in iKOs [149]. These data suggest that some of the cardiovascular phenotypes associated with *Bmal1* depletion may result from *Bmal1* function during development. Although it is clear that there is a link between the circadian clock and atherosclerosis, further dissection of the importance of BMAL1 and other clock proteins in this disease is warranted.

## Circadian rhythms in the nervous system

Circadian rhythms in the suprachiasmatic nucleus (SCN) have been the focus of many years of research; but how the SCN imposes rhythmicity throughout the body (or even locally in the brain) is not fully understood. Recent studies have broadened the focus from neurons to astrocytes, demonstrating the important role of these glial cells in maintaining circadian rhythmicity [150–152]. A recent circadian atlas of non-human primates includes 64 tissues in the body, including 22 different regions in the brain [153]. The authors found genes cycling across the day in all brain regions, providing a comprehensive view of the reach of the circadian clock throughout the CNS of baboons [153]. While further studies are needed to understand fully the impact of rhythms in the nervous system and all their potential functions, the following studies are a step in that direction.

### Circadian rhythms in the blood–brain barrier

The blood–brain barrier (BBB) is highly selective as to what it allows into the brain, and its permeability is regulated (in part) by the circadian clock. Mice that lack *Bmal1* in both the CNS and peripheral nervous system exhibit BBB hyperpermeability with an age-dependent loss of pericyte coverage of blood vessels in the brain [154], suggesting that the circadian clock regulates BBB homeostasis. Consistent with this, the *Drosophila* ‘BBB’ is more permeable at night [155]. The fly ‘BBB’ consists of a layer of subperineurial glia and perineurial glia that surrounds the entire CNS. Zhang et al. [155] showed that at night, the perineurial glia clock increases gap junctions and lowers Mg^2+^ levels, reducing transporter efflux activity; thus, xenobiotics are taken up by the brain. As passage through the BBB is necessary for the treatment of CNS diseases, these findings may have very practical applications. In a proof-of-principle experiment, Zhang and colleagues [155] also demonstrated that an anti-seizure drug is more effective when administered at night. Altogether, as the insect and mammalian BBB share many structural and functional similarities, this is a potentially major finding for human physiology.

### Effects of light in mood and learning

Light is a strong external signal for the circadian system [156]. Its detection involves three classes of photoreceptors in the retina: classic rods and cones, and a subset of retinal ganglion cells (RGCs) that express the photopigment melanopsin (*Opn4*), which makes them intrinsically photosensitive (ipRGCs) [25–27]. When ipRGCs are lost, the SCN no longer receives light information. Unexpectedly, ablation of these photoreceptors in mice affects mood and learning, indicating that ipRGCs are sensory cells that drive these behavioral effects [156]. Fernandez et al. [157] recently showed that these neurons relay light information that influences cognitive functions in an SCN-independent manner. Instead, an SCN-independent subset of ipRGCs connects to the perihabenular nucleus of the thalamus [157]. In summary, there appear to be two distinct retina–brain pathways that integrate light and highlight its influence on learning and mood. This takes us one step closer to research aiming to investigate light modulation as a potential strategy for treating mood disorders.

## Circadian disruption in cancer

Epidemiological studies have linked circadian disruption to increased cancer susceptibility in all key organ systems [158–160]. Compelling evidence has shown that polymorphisms in the core circadian genes *Per1*, *Per2*, and *Per3* are frequently found in human cancers, resulting in decreased expression of these genes [158], and that oncogenic MYC suppresses the clock [161]. Genetic loss of *Per2* or *Bmal1* promotes lung tumorigenesis in mice, leading to increased c-Myc expression, enhanced proliferation, and metabolic dysregulation [50]. Similarly, hepatocellular carcinoma (HCC) is induced by chronic jet lag in mice in a manner similar to that observed in obese humans: beginning with non-alcoholic fatty liver disease (NAFLD), then progressing to steatohepatitis and fibrosis and, ultimately, to HCC [49] (Fig.3). Thus, these two studies have convincingly shown a mechanistic connection between clock disruption and cancer development [49, 50]. In addition, microRNA miR-211, which suppresses *Clock* and *Bmal1*, also promotes tumor progression [162]. Targeting REV-ERBs is an effective strategy for combating cancer without altering the viability of normal cells or tissues. Using anticancer agonists of REV-ERBs (SR9009 and SR9011), Sulli et al. [58] were able to interfere with at least two cancer hallmarks: de novo lipogenesis and autophagy, which are important in meeting the metabolic demands of cancer cells.

Low oxygen levels in solid tumors stabilize hypoxia-inducible factors (HIFs), which are transcription factors that acidify the tumor microenvironment. Recent research has shown that HIFs are capable of influencing various clock transcripts [163–165]. Furthermore, Walton et al. [166] showed that acidfication of the tumor microenvironment by hypoxic cells disrupts the circadian clock and rhythmic transcriptome. They showed that low pH suppresses mTORC1 (Mammalian target of rapamycin complex 1) signaling, causing inhibition of translation. The authors further found that restoring mTORC1 signaling, either by buffering against acidification or by inhibiting lactic acid production, fully rescues translation and clock oscillations [166]. Overall, recent research on circadian rhythms and cancer has given major insights into disease mechanisms, which will hopefully allow for improved treatments, possibly including circadian considerations.

## Circadian rhythms in aging

Circadian rhythms seem to decline with age [167, 168], with neuronal activity rhythms displaying an age-dependent decline in the master clock in the SCN [169]. In addition, the disruption of circadian rhythms through the ablation of *Bmal1* leads to premature aging in mice [84]. Recent studies of aged stem cells and liver suggest that circadian transcriptional profiles in aging cells are rewired. However, in contrast with what was predicted, aging does not simply cause dampened circadian rhythmicity in the expression of genes that cycle when animals are young. Instead, a new set of genes begin to cycle in aged mice [83, 170]. Aged epidermal and skeletal muscle stem cells show reprogramming of gene expression towards a stress response, inflammation, and DNA damage, with core clock genes maintaining their rhythms [83]. Thus, this study supports the idea that aged stem cells retain a functional clock, but that this clock redirects the cell into new circadian functions with age. Perhaps this reprogramming is associated with the differential DNA methylation that occurs with aging [171] (see below). The key pathways or molecules that lead to this rewiring of the circadian transcriptome with aging remain unknown.

Additional studies have brought to light extra layers of circadian regulation that appear to decline with age. Polyamines modulate multiple cellular functions, and altered polyamine metabolism is associated with aging. Zwighaft et al. [55] linked polyamine metabolism, the clock, and aging, showing that the circadian clock controls polyamine levels and, in turn, that polyamines regulate circadian period. Polyamines exert their effects by impacting the interaction between the circadian repressors PER2 and CRY1. Interestingly, the longer circadian period of aged mice can be shortened with polyamine supplementation in the drinking water [55]. Another layer of circadian regulation appears to be in the modification of cytosines in DNA. De novo DNA methylation is established by the DNA methyltransferases DNMT3A and DNMT3B, which transfer a methyl group from S-adenosylmethionine to a cytosine at a cytosine guanine (CpG) site. On the other hand, cytosine methylation marks can be removed through an active demethylation pathway involving oxidation performed by TET (ten eleven translocation) enzymes [171]. DNA methylation might affect gene regulation by changing nucleosome stability and altering the nucleosome structure. Recently, Oh et al. [172] reported that a large proportion of cytosines show a circadian pattern of methylation in mice, and that the mRNA levels of nearby genes are positively correlated with corresponding oscillations in DNA methylation in liver and lung tissues. Consistent with the decrease of circadian oscillation of certain transcripts with age, oscillatory cytosine modifications (and DNA methylation, in general) also appear to decrease in older animals [172].

Alzheimer’s disease (AD) patients frequently experience increased daytime sleep and night-time wakefulness [54]. *AD is associated with* the production and deposition of the *β*-*amyloid* (Aβ) peptide, and soluble Aβ levels exhibit robust daily oscillations in mouse hippocampal interstitial fluid [78, 173]. However, little is known about how circadian rhythms may influence AD [174]. In a recent study trying to address the role of the circadian clock in determining Aβ levels, Kress et al. [175] showed that Aβ rhythms are normal when *Bmal1* is deleted in the brain and retained only in the SCN. Nevertheless, whole-brain *Bmal1* deletion causes loss of Aβ interstitial fluid rhythms in the hippocampus and markedly increases amyloid plaque burden. In addition to Aβ oscillations, tau levels also fluctuate in the brain interstitial fluid of mice and in the cerebral spinal fluid (CSF) of humans [54]. Tau levels appear to be higher during the animal’s active period and to increase when animals are subjected to sleep deprivation. Similarly, human CSF tau levels also increased by over 50% during sleep deprivation [54]. Finally, an interesting human cross-sectional study revealed an association between pre-clinical AD and disruption of the activity–rest rhythms. Specifically, pre-clinical amyloid plaques or higher CSF phosphorylated-tau to Aβ-42 ratios were associated with increased variability in daily behaviors, indicating fragmentation of activity–rest rhythms. The presence of abnormalities in circadian rhythms in pre-clinical AD suggests that circadian dysfunction could contribute to early pathogenesis or could serve as a biomarker of AD [176]. Together, these studies suggest that we should investigate the importance of a healthy sleep–wake cycle as an intervention for preventing AD and other tauopathies.

## Implications for translation to therapy

Circadian research, in particular the concept of chrono-pharmacology, is increasingly shaping our view of future research and medicine [177, 178]. It has introduced a time component to our view of metabolism, inflammation, and host–pathogen interactions (among other interactions), and has shown that targeting genes that are cycling at specific times of day may be advantageous [179–181]. Recent characterizations of the circadian transcriptional profiles of non-human primates [153] and humans [46] across multiple tissues have complemented the circadian atlas previously obtained for mice [181]. These reports have strengthened an important conclusion from the rodent data—the potential for chrono-pharmacological treatment of multiple diseases. Most of the protein-coding genes that have been found to be oscillating in primates encode proteins that are identified as druggable targets by the US Food and Drug Administration.

Regarding infectious diseases, treatments, and vaccinations could be more effective when administered at specific times of day. Indeed, influenza vaccine administration in the morning has been shown to improve antibody response over afternoon vaccination response in people over 65 years old [182]. This shows the potential to align the timing of external interventions, such as drug treatment or vaccinations, to the phase of our internal defenses. A further aspect to take into consideration is the potential for the pathogen itself to have circadian rhythms, as is the case for the sleeping sickness parasite, *Trypanosoma brucei.* We recently showed that this parasite has intrinsic circadian rhythms that affect its sensitivity to suramin treatment [183]. This may be a common feature of pathogens, although this remains to be determined.

Pharmacological modulation of the circadian machinery may also be an effective therapy for cancer [58] and potentially for sleep and anxiety [184]. Our own studies on parasite–host interactions may help to identify factors that alter the period of the circadian clock [138]. If so, molecule(s) could potentially be used to accelerate the rhythms of both central and/or peripheral clocks, helping people to overcome jet lag or even improving symptoms in patients with DSPD. The fact that physiology is intimately linked with circadian rhythmicity raises the question of when to intervene in all human diseases, and if there is a particular time of the day when treatment would be more effective or whether modulating a key clock protein function could alleviate the pathology.

## Conclusions and future directions

The past few years have been very exciting for circadian research, making it clear that circadian biology is at the core of animal physiology. A multitude of additional layers of circadian clock regulatory mechanisms have been demonstrated recently. Such additional layers of regulation of the circadian clock machinery include chromatin conformation and interactions [43, 56], polyamines [55], NADP^+^:NADPH redox ratio [185], cytosine modifications [172], and even autophagy [120]. Among those, the genomics of circadian rhythms have expanded our understanding of daily physiological rhythms in health [43, 88, 112] and disease [53, 162].

In addition to circadian rhythms, there are also biological rhythms with shorter (ultradian) periods. Clusters of ultradian genes that cycle with a 12-h period have been identified in several peripheral tissues in mice [181, 186], many of which respond to feeding [187]. Recently, it was proposed that the mechanism behind these 12-h rhythms is a cell-autonomous 12-h pacemaker that is important for maintaining metabolic homeostasis [188]. In the future, it will be interesting to see what other aspects of physiology are influenced by ultradian rhythms and how they integrate with circadian physiology.

Overall, we believe that the growing body of evidence in mammalian circadian rhythms research is revealing an undisputable link between circadian rhythms and human health. Nevertheless, we are far from understanding the complexity of circadian biology and medicine. Exciting new aspects continue to emerge in terms of health and lifespan, including dietary influences [189], as well as differences between genders [190]. Circadian medicine is clearly an interdisciplinary field that requires complementary expertise [57, 138, 175]. Advances in technology have shaped circadian research in recent years [43, 73, 112] and will continue to be crucial going forward. Integrating the temporal axis into human physiology and medicine offers an opportunity to optimize the alignment of our internal rhythms to the environment, which will provide new opportunities for lifestyle and pharmacological interventions to treat diseases and promote health.

## Supplementary information

**Additional file 1: Table S1.** Summary of advances in studies of the different layers of regulation of circadian gene expression on a genome-wide scale.

## Abbreviations

AD: Alzheimer’s disease
Aβ: Amyloid β
BBB: Blood–brain barrier
CNS: Central nervous system
Cry1: Cryptochrome 1
CSF: Cerebrospinal fluid
DHS: DNase hypersensitive site
DIO: Diet-induced obesity
DSPD: Delayed sleep phase disorder
FASPD: Familial advanced sleep phase disorder
HCC: Hepatocellular carcinoma
HIF: Hypoxia-inducible factor
iKO: Inducible knockout
IL-7R: Interleukin-7 receptor
ipRGC: Intrinsically photosensitive RGC
mTORC1: Mammalian target of rapamycin complex 1
NOB: Nobilitin
Pol II: RNA polymerase II
PPARα: Peroxisome proliferated activated receptor alpha
RGC: Retinal ganglion cell
SCN: Suprachiasmatic nucleus
SIK3: Salt-inducible kinase 3
TSS: Transcription start site
