# Supplementary material for: Genomics of circadian rhythms in health and disease
Source: Genome Med. 2019 Dec 17;11:82. doi: 10.1186/s13073-019-0704-0 (PMC6916512; doi:10.1186/s13073-019-0704-0)
Supplement: Supplementary file 1 — Additional file 1: Table S1. Summary of advances in studies of the different layers of regulation of circadian gene expression on a genome-wide scale. [file 13073_2019_704_MOESM1_ESM.docx]

**Table S1** – Summary of advances in studies of the different layers of circadian gene expression regulation on a genome-wide scale.

| Major circadian genome-wide regulatory layer | Mammal | Tissue & Experimental design | Technical approach |
| --- | --- | --- | --- |
| Circadian transcriptome | Mouse | Liver, 4-hr resolution, 2 cycles, DD [1, 2], 3-hr resolution, 1 cycle, DD [3], 4-hr resolution, 1 cycle, LD [4]  Multiple tissues, 2-6-hr resolution, 2 cycles, DD [5] | Microarray [1, 5-7];  RNA-sequencing [2, 3, 5];  Nascent-Seq [4] |
|  | Non-human primate | Multiple tissues, 2h resolution, 1 cycle [8] | RNA-sequencing |
|  | Human | Blood, 3-hr resolution, 1 cycles, 26 subjects [9]; 4-hr resolution, 1 cycles, 22 subjects [10]  Brain: Cortical and limbic regions (uneven sampling – time of death 55 subjects) [11]  Prefrontal cortex (uneven sampling – time of death from 146 subjects) [12]  Liver and lung (uneven sampling – time of death) [13]  Subcutaneous adipose tissue (6-hr resolution, 1.25 cycle, 7 subjects) [14]  Multiple tissues (uneven sampling – time of death from 632 subjects) [15]  Skeletal muscle (4-hr resolution, 1 cycle, 10 subjects) [16] | Microarray  RNA-sequencing [15, 16] |
| Circadian transcription factor binding | Mouse | Liver, 4-hr resolution, 1 cycle, LD [17, 18], 4-hr resolution, 2 cycles, DD [2], 1-time point [4]. 2-time points LD [19]  Pancreatic islet β cell line [20] | Chromatin immunoprecipitation-seq (ChIP-seq) of:  BMAL1 [17];  BMAL1 and CLOCK [4];  BMAL1, CLOCK, NPAS2, PER1, PER2, CRY1, CRY2 [2];  BMAL1 and CLOCK [20]  REV-ERBa/b [18]  DBP [19] |
| Histone modification rhythmicity | Mouse | Liver, 4-hr resolution, 2 cycles, DD [2], 3-hr resolution, 1 cycle, DD [3], 4-hr resolution, 1 cycle, LD, night-restricted feeding [21]  Pancreatic islet β cell line [20] | ChIP-seq of histone modifications H3K4me1, H3K4me3, H3K9ac, H3K27ac, H3K36me3 and H3K79me2 [2];  H3K4me1, H3K4me3, H3K9ac, H3K27ac, and H3K36me3 [3];  H3K4me3, H3K36me3 [21];  H3K4me2, H3K27ac, H2AZ [20] |
| Adenosine-to-inosine (A-to-I) RNA-editing rhythms | Mouse | Liver, 4-hr resolution, 2 cycles [22] | Direct sequencing |
| Circadian RNA Polymerase II recruitment | Mouse | Liver, 4-hr resolution, 2 cycles, DD [2];  4-hr resolution, 1 cycle, LD, night-restricted feeding [21] | ChIP-seq of p300, RNA Pol II, CBP (Koike 2012);  RNA Pol II [21] |
| Circadian enhancer activity | Mouse | Liver, 3-hr resolution, 1 cycle, LD | Global Run-On sequencing (GRO-seq) [23] |
| Circadian chromatin interactions at the *Dbp* locus | Mouse | Mouse embryonic fibroblasts (4-hr resolution, 1 cycle) | Chromosome conformation capture on chip (4C, also known as 3C on chip, one-to-all) [24] |
| Circadian chromatin mobility between sub-nuclear compartments | Human | Human embryonic stem cells and derived embryoid bodies  (4-hr resolution, 2 cycles) | in situ proximity ligation assay (ISPLA) [25] |
| Circadian chromatin interactions with master transcriptional regulators | Human | Human embryonic stem cells and derived embryoid bodies (4-hr resolution, 2 cycles) [25] | Circular chromosome conformation capture sequencing (4C-seq, also known as circular 3C, one-to-all) PARP1and CTCF [25] |
|  | Mouse | Liver, 12-hr resolution, 1 cycle, DD [26]  Liver and kidney (12-hr resolution, 1 cycle, LD) | Cohesin and CTCF [26]  Cry1 and Glycogen Synthase 2 (Gys2) [27] |
| Circadian chromatin accessibility | Mouse | Liver, 4-hr resolution, 1 cycle, LD | DNase I hypersensitive site mapping [28] |
| Tissue-specific chromatin interactions | Mouse | Liver and kidney (1 time point) | 4C-seq [29] |
| Circadian chromatin interactions via Pol II | Mouse | Liver (12-hr resolution, 1 cycle, LD) | Chromatin Interaction Analysis by Paired-End Tag Sequencing (ChIA-PET) [30] |
| Genome-wide circadian chromosomal interactions | Human | Human fibroblasts (8-hr resolution, 2.5 cycles) [31] | Chromosome conformation capture (Hi-C, all-to-all) |
|  | Mouse | Liver (12-hr resolution, 1 cycle, LD) [32] |  |

**Note:**

LD – tissue collection was performed under Light/Dark conditions

DD – tissue collection was performed under constant darkness

**References:**

1. Panda, S., et al., *Coordinated transcription of key pathways in the mouse by the circadian clock.* Cell, 2002. **109**(3): p. 307-20.

2. Koike, N., et al., *Transcriptional Architecture and Chromatin Landscape of the Core Circadian Clock in Mammals.* Science, 2012.

3. Vollmers, C., et al., *Circadian Oscillations of Protein-Coding and Regulatory RNAs in a Highly Dynamic Mammalian Liver Epigenome.* Cell Metab, 2012. **16**(6): p. 833-45.

4. Menet, J.S., et al., *Nascent-Seq reveals novel features of mouse circadian transcriptional regulation.* Elife, 2012. **1**: p. e00011.

5. Zhang, R., et al., *A circadian gene expression atlas in mammals: implications for biology and medicine.* Proc Natl Acad Sci U S A, 2014. **111**(45): p. 16219-24.

6. Ueda, H.R., et al., *A transcription factor response element for gene expression during circadian night.* Nature, 2002. **418**(6897): p. 534-9.

7. Storch, K.F., et al., *Extensive and divergent circadian gene expression in liver and heart.* Nature, 2002. **417**(6884): p. 78-83.

8. Mure, L.S., et al., *Diurnal transcriptome atlas of a primate across major neural and peripheral tissues.* Science, 2018. **359**(6381).

9. Moller-Levet, C.S., et al., *Effects of insufficient sleep on circadian rhythmicity and expression amplitude of the human blood transcriptome.* Proc Natl Acad Sci U S A, 2013. **110**(12): p. E1132-41.

10. Archer, S.N., et al., *Mistimed sleep disrupts circadian regulation of the human transcriptome.* Proc Natl Acad Sci U S A, 2014. **111**(6): p. E682-91.

11. Li, J.Z., et al., *Circadian patterns of gene expression in the human brain and disruption in major depressive disorder.* Proc Natl Acad Sci U S A, 2013. **110**(24): p. 9950-5.

12. Chen, C.Y., et al., *Effects of aging on circadian patterns of gene expression in the human prefrontal cortex.* Proc Natl Acad Sci U S A, 2016. **113**(1): p. 206-11.

13. Anafi, R.C., et al., *CYCLOPS reveals human transcriptional rhythms in health and disease.* Proc Natl Acad Sci U S A, 2017. **114**(20): p. 5312-5317.

14. Christou, S., et al., *Circadian regulation in human white adipose tissue revealed by transcriptome and metabolic network analysis.* Sci Rep, 2019. **9**(1): p. 2641.

15. Ruben, M.D., et al., *A database of tissue-specific rhythmically expressed human genes has potential applications in circadian medicine.* Sci Transl Med, 2018. **10**(458).

16. Perrin, L., et al., *Transcriptomic analyses reveal rhythmic and CLOCK-driven pathways in human skeletal muscle.* Elife, 2018. **7**.

17. Rey, G., et al., *Genome-wide and phase-specific DNA-binding rhythms of BMAL1 control circadian output functions in mouse liver.* PLoS Biol, 2011. **9**(2): p. e1000595.

18. Cho, H., et al., *Regulation of circadian behaviour and metabolism by REV-ERB-alpha and REV-ERB-beta.* Nature, 2012. **485**(7396): p. 123-7.

19. Yoshitane, H., et al., *Functional D-box sequences reset the circadian clock and drive mRNA rhythms.* Commun Biol, 2019. **2**: p. 300.

20. Perelis, M., et al., *Pancreatic beta cell enhancers regulate rhythmic transcription of genes controlling insulin secretion.* Science, 2015. **350**(6261): p. aac4250.

21. Le Martelot, G., et al., *Genome-wide RNA polymerase II profiles and RNA accumulation reveal kinetics of transcription and associated epigenetic changes during diurnal cycles.* PLoS Biol, 2012. **10**(11): p. e1001442.

22. Terajima, H., et al., *ADARB1 catalyzes circadian A-to-I editing and regulates RNA rhythm.* Nat Genet, 2017. **49**(1): p. 146-151.

23. Fang, B., et al., *Circadian enhancers coordinate multiple phases of rhythmic gene transcription in vivo.* Cell, 2014. **159**(5): p. 1140-52.

24. Aguilar-Arnal, L., et al., *Cycles in spatial and temporal chromosomal organization driven by the circadian clock.* Nat Struct Mol Biol, 2013. **20**(10): p. 1206-13.

25. Zhao, H., et al., *PARP1- and CTCF-Mediated Interactions between Active and Repressed Chromatin at the Lamina Promote Oscillating Transcription.* Mol Cell, 2015. **59**(6): p. 984-97.

26. Xu, Y., et al., *Long-Range Chromosome Interactions Mediated by Cohesin Shape Circadian Gene Expression.* PLoS Genet, 2016. **12**(5): p. e1005992.

27. Mermet, J., et al., *Clock-dependent chromatin topology modulates circadian transcription and behavior.* Genes Dev, 2018. **32**(5-6): p. 347-358.

28. Sobel, J.A., et al., *Transcriptional regulatory logic of the diurnal cycle in the mouse liver.* PLoS Biol, 2017. **15**(4): p. e2001069.

29. Yeung, J., et al., *Transcription factor activity rhythms and tissue-specific chromatin interactions explain circadian gene expression across organs.* Genome Res, 2018. **28**(2): p. 182-191.

30. Beytebiere, J.R., et al., *Tissue-specific BMAL1 cistromes reveal that rhythmic transcription is associated with rhythmic enhancer-enhancer interactions.* Genes Dev, 2019. **33**(5-6): p. 294-309.

31. Chen, H., et al., *Functional organization of the human 4D Nucleome.* Proc Natl Acad Sci U S A, 2015. **112**(26): p. 8002-7.

32. Kim, Y.H., et al., *Rev-erbalpha dynamically modulates chromatin looping to control circadian gene transcription.* Science, 2018. **359**(6381): p. 1274-1277.
